# Developmental trajectories of gyrification and sulcal morphometrics in children and adolescents at high familial risk for bipolar disorder or schizophrenia

**DOI:** 10.1016/j.dcn.2025.101536

**Published:** 2025-02-24

**Authors:** Simon R. Poortman, Jakub Jamarík, Louise ten Harmsen van der Beek, Nikita Setiaman, Manon H.J. Hillegers, Marjolein E.A. Barendse, Neeltje E.M. van Haren

**Affiliations:** a Department of Child and Adolescent Psychiatry/Psychology, Erasmus University Medical Center, Sophia Children’s Hospital, Rotterdam, the Netherlands; b Faculty of Medicine, Masaryk University, Brno, Czech Republic; c Department of Psychiatry, University Medical Center Utrecht Brain Center, Utrecht, the Netherlands

**Keywords:** Bipolar disorder, Gyrification, High familial risk, Longitudinal, Offspring, Schizophrenia

## Abstract

Offspring of parents with severe mental illness are at increased risk of developing psychopathology. Identifying endophenotypic markers in high-familial-risk individuals can aid in early detection and inform development of prevention strategies. Using generalized additive mixed models, we compared age trajectories of gyrification index (GI) and sulcal morphometric measures (i.e., sulcal depth, length and width) between individuals at familial risk for bipolar disorder or schizophrenia and controls. 300 T1-weighted MRI scans were obtained of 187 individuals (53 % female, age range: 8–23 years) at familial risk for bipolar disorder (n = 80, n families=55) or schizophrenia (n = 53, n families=36) and controls (n = 54, n families=33). 113 individuals underwent two scans. Globally, GI, sulcal depth and sulcal length decreased significantly with age, and sulcal width increased significantly with age in a (near-)linear manner. There were no differences between groups in age trajectories or mean values of gyrification or any of the sulcal measures. These findings suggest that, on average, young individuals at familial risk for bipolar disorder or schizophrenia have preserved developmental patterns of gyrification and sulcal morphometrics during childhood and adolescence.

## Highlights

• Gyrification, and sulcal depth and length decrease with age during adolescence.
• Sulcal width increases with age during adolescence.
• Age trajectories of brain morphometrics were mostly (near-)linear.
• Young high-familial-risk individuals show preserved age trajectories relative to controls.
• Controlling for IQ or symptom severity did not change study findings.

## Graphical Abstract

**Figure:**
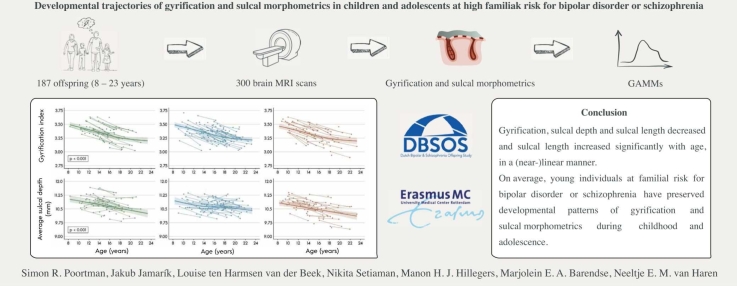

## 1 Introduction

Schizophrenia (SZ) and bipolar disorder (BD) are neurodevelopmental disorders with high heritability rates and shared genetic-risk loci (Hilker et al., 2018, Palaniyappan and Liddle, 2014, Ruderfer et al., 2018). Child and adolescent offspring of parents with SZ (SZo) or BD (BDo) are at increased familial risk of developing mental illness themselves (Dean et al., 2010, Gottesman et al., 2010, Lau et al., 2018, Mesman et al., 2013, Rasic et al., 2014, Uher et al., 2023). They represent a valuable population to study as they are often still unmedicated and in a sensitive developmental period for the onset of psychiatric symptoms (Dalsgaard et al., 2020, Kessler et al., 2005). By longitudinally following high-familial-risk offspring during adolescent development, we aim to identify endophenotypic markers that may inform increased risk or imply mechanisms that underlie the intergenerational transmission of risk for severe mental illness. Such markers will benefit early detection and may inform preventive intervention, which are essential for managing symptoms and improving long-term outcomes (Anderson et al., 2018, Correll et al., 2018, Fusar-Poli et al., 2021, McGorry and Nelson, 2016, Shah et al., 2022, Uhlhaas et al., 2023).

**Fig. 1:**
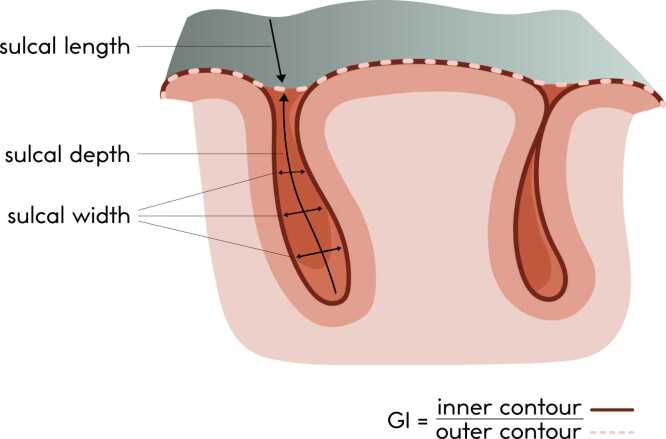
Schematic representation of the gyrification index and sulcal morphometrics used in the current study. The gyrification index (GI) is defined as the cortical surface area (inner contour, solid line) divided by the hull surface area (outer contour, dashed line). Sulcal depth is calculated as the geodesic distance between the sulcal base and the hull, averaged across all points along the median sulcal surface. Sulcal length is determined on the hull and defined as the summed distance of the intersections between the median sulcal surfaces and the hull. Sulcal width is calculated as the width of the CSF in the sulcus: it is the volume of cerebrospinal fluid in the sulcus, divided by the surface of the skeleton mesh. Adapted with permission from “Longitudinal Allometry of Sulcal Morphology in Health and Schizophrenia” by Janssen et al., (2022), *The Journal of Neuroscience*, *42(18)*, p. 3706.

Cortical gyrification, the remodeling of brain surface morphology to create sulci and gyri and thereby expanding the cortical surface area (White et al., 2010), has emerged as a promising endophenotypic marker for psychosis and risk for SZ and BD (Bois et al., 2015, Choi et al., 2022, Falkai et al., 2007, Madeira et al., 2020, Madre et al., 2020). The gyrification index (GI), which is the ratio of the pial and outer hull perimeters typically obtained using structural T1-weighted magnetic resonance imaging (MRI), can be used to quantify this marker (Schaer et al., 2008, Zilles et al., 1988) (Fig. 1). In adolescence, the GI and sulcal depth decrease with age, while sulcal width is observed to increase with age, which is consistent with the understanding that the brain undergoes synaptic pruning during this period, a process during which redundant neural connections are eliminated (Alemán-Gómez et al., 2013, White et al., 2010).

Studies show that individuals with SZ and BD exhibit significantly different GI patterns than controls, involving mostly prefrontal, parietal and temporal areas (Miola et al., 2022, Sasabayashi et al., 2021). However, these findings are inconsistent in their direction and show both hypo- and hypergyrification. Based on a recent review, adolescents and young adults with SZ mostly show frontal and widespread hypergyrification, while in their mid-thirties and above they generally show frontal and widespread hypogyrification or decreased sulcation. Fewer studies have compared GI in BD and controls, with a particular paucity of studies in adolescents and young adults. In adult studies, both increased frontal curvature and reduced frontal GI or sulcation have been found (Miola et al., 2022, Sasabayashi et al., 2021). Regarding sulcal morphometry, increased sulcal width was found in adolescents with SZ and BD compared to controls, frontally in those with BD and in all lobes in those with SZ, while no differences were observed in GI (Janssen et al., 2014). Longitudinal age trajectories through adulthood show a steeper decrease of sulcation and sulcal area, but not sulcal depth or length, in individuals with SZ compared to controls (Janssen et al., 2022).

Studies investigating GI in individuals at (familial) risk for SZ and BD are few and limited to mostly adults and low sample sizes. Widespread hypergyrification, especially (pre)frontal and parietal, was found in adult individuals at familial risk for SZ (Falkai et al., 2007), adolescents and young adults at risk for psychosis for non-familial reasons (e.g., cognitive reasons, at-risk mental state) (Sasabayashi et al., 2017, Stanfield et al., 2008, Tepest et al., 2013) (but see (Jou et al., 2005; Nanda et al., 2014)), and familial and clinical high-risk adolescents and young adults that converted to psychosis compared to those that did not (Basavaraju et al., 2023, Harris et al., 2007, Harris et al., 2004). No differences in GI were found in adolescent and young adult individuals at high familial risk for BD (Drobinin et al., 2019, Nanda et al., 2014). To our knowledge, there are currently no longitudinal studies that have investigated developmental trajectories of GI or sulcal depth, length or width in child and adolescent SZo and BDo.

The aim of this prospective, longitudinal, cross-disorder study is threefold. First, we investigate GI trajectories over time in SZo and BDo compared to controls. Based on previous studies in case-control and high-risk samples, we postulate that particularly (pre)frontal and parietal GI trajectories of SZo differ significantly from control offspring, showing hypergyrification across the entire age range, and that this effect will be attenuated when controlling for psychosis symptoms. Secondly, age trajectories of sulcal depth, length and width are examined to acquire a more comprehensive understanding of cortical folding processes, alongside gyrification, in children and adolescents at familial risk for SZ and BD. Drawing on prior research (Janssen et al., 2014), we hypothesize that sulcal width increases to a larger extent during adolescence in SZo and BDo compared with controls. Third, we will determine whether there were group differences in whether a sulcus was successfully identified or not. In some individuals, a sulcus may not be present or is, due to its shape or form, unmeasurable by the software.

## 2 Methods

### 2.1 Participants

The Dutch Bipolar and Schizophrenia Offspring Study (DBSOS) is an ongoing prospective cohort study, investigating the development of the brain, genetics, cognitive functioning, and environment, that contribute to risk for and resilience against mental illness (Setiaman et al., 2023). Data from the first two waves is used for the current study. After exclusions for scan quality and other reasons (see Supplement 1.1), the current sample includes a total of 300 T1-weighted MRI scans of 187 child, adolescent and young adult individuals at high familial risk (of whom 96 % were offspring) (80 BDo from 55 families, 53 SZo from 36 families and 54 controls from 33 families) from 124 families. Wave 1 included 140 individuals aged between 8 and 18 years (54 BDo, 42 SZo and 44 controls). Wave 2 comprised 160 individuals aged between 11 and 23 years (72 BDo, 43 SZo and 45 controls). A total of 113 individuals (46 BDo, 32 SZo and 35 controls) were scanned at both waves, with 2.2–5.9 years between assessments (mean=3.9 years, Table 1; see SFigure S1 for the age scatter plot).

**Table 1:** Demographic and clinical characteristics.

	Bipolar Disorder offspring		Schizophrenia offspring		Control offspring		Main group effect				Pairwise (*p* < .05)	
	Wave 1	Wave 2	Wave 1	Wave 2	Wave 1	Wave 2	Wave 1		Wave 2		Wave 1	Wave 2
							*F*	*p*	*F*	*p*		
*n*	54	72	42	43	44	45						
*n* Families	40	51	30	30	29	29						
Age at scan, years, mean (SD)	14.12 (2.57)	17.89 (2.56)	13.17 (2.89)	16.68 (2.95)	13.33 (2.16)	16.75 (2.45)	1.95	0.146	3.92	**0.022**		BDo > SZo
Sex, F/M, *n* (% female)	26/28 (48)	35/37 (49)	25/17 (60)	32/11 (74)	20/24 (45)	22/23 (49)		0.380		**0.014**		SZo > BDo & Co
IQ, mean (SD)	106.3 (19.1)	104.6 (14.3)	102.3 (19.0)	100.5 (19.1)	116.6 (12.5)	114.0 (12.9)	7.92	**< 0.001**	9.15	**< 0.001**	BDo & SZo < Co	BDo & SZo < Co
Single scan, *n*	8	26	10	11	9	10						
Both scans, *n*	46	46	32	32	35	35						
Scan interval, years, mean (SD)^a^	3.99 (0.69)		3.81 (0.62)		3.70 (0.98)		*F* = 1.40, *p* = 0.251					
DSM-IV diagnosis, *n* (%)	27 (50)	45 (63)	22 (52)	31 (72)	9 (20)	12 (27)		**0.002**		**< 0.001**	BDo & SZo > Co	BDo & SZo > Co
K-SADS sum sscores, mean (SD)												
Depression	39.57 (9.49)	45.00 (12.12)	39.60 (9.99)	45.37 (11.85)	33.95 (3.73)	36.82 (7.65)	6.92	**0.001**	9.31	**< 0.001**	BDo & SZo > Co	BDo & SZo > Co
Mania	10.37 (3.43)	10.89 (3.34)	9.57 (1.25)	10.28 (2.74)	9.07 (0.33)	9.11 (0.38)	4.19	**0.017**	6.19	**0.003**	BDo > Co	BDo > Co
Psychosis^b^	34.72 (2.77)	36.14 (4.11)	35.74 (3.57)	36.53 (3.53)	33.45 (0.87)	33.71 (1.62)	8.04	**< 0.001**	9.33	**< 0.001**	SZo > Co	BDo & SZo > Co
DSM-IV diagnosis, *n* (%)												
No diagnosis	27 (50.0)	27 (37.5)	20 (47.6)	12 (27.9)	35 (79.5)	33 (73.3)		**0.002**		**< 0.001**	BDo & SZo < Co	BDo & SZo < Co
Developmental disorder^c^	8 (14.8)	20 (27.8)	10 (23.8)	17 (39.5)	1 (2.3)	6 (13.3)		**0.008**		**0.018**	BDo & SZo > Co	SZo > Co
Anxiety disorder^d^	6 (11.1)	11 (15.3)	7 (16.7)	9 (20.9)	2 (4.5)	3 (6.7)		0.189		0.146		
Mild mood disorder^e^	17 (31.5)	22 (30.6)	2 (4.8)	5 (11.6)	4 (9.1)	4 (8.9)		**0.001**		**0.006**	BDo > SZo & Co	BDo > SZo & Co
Major depressive disorder	1 (1.9)	10 (13.9)	5 (11.9)	7 (16.3)	0 (0.0)	0 (0.0)		**0.018**		**0.008**	SZo > Co	BDo & SZo > Co
Manic^f^	2 (3.7)	2 (2.8)	0 (0.0)	1 (2.3)	0 (0.0)	0 (0.0)		0.333		0.620		
Psychotic	0 (0.0)	1 (1.4)	0 (0.0)	0 (0.0)	0 (0.0)	0 (0.0)				1.000		
Substance use disorder^g^	2 (3.7)	7 (9.7)	1 (2.4)	3 (7.0)	0 (0.0)	0 (0.0)		0.636		0.082		
Other	7 (13.0)	15 (20.8)	8 (19.0)	8 (18.6)	4 (9.1)	5 (11.1)		0.409		0.399		
Psychotropic medication^h^, *n* (%)	5 (9)	15 (21)	0 (0)	9 (21)	0 (0)	4 (9)		**0.012**		0.189	NS	

Statistical comparison was performed using Fisher’s exact test for categorical and analyses of variance (Tukey’s test for pairwise comparisons) for continuous variables.

BDo = bipolar disorder offspring, Co = control offspring, K-SADS = Schedule for Affective Disorders and Schizophrenia for School-Age Children, NS = not significant, SZo = schizophrenia offspring.

**bold** = statistically significant (*p* ≤ 0.05).

a 113 (46 bipolar disorder offspring, 32 schizophrenia offspring and 35 control offspring) of 187 offspring (60 %) were scanned at both time points.

b Data missing for one bipolar disorder offspring at wave 1.

c Developmental disorders include attention-deficit/hyperactivity disorder, autism spectrum disorder (including Asperger syndrome and childhood disintegrative disorder), conduct disorder, disruptive behavior disorder not otherwise specified and oppositional defiant disorder.

d Anxiety disorders include acute stress disorder, adjustment disorder with anxiety, agoraphobia, anxiety disorder not otherwise specified, generalized anxiety disorder, obsessive-compulsive disorder, panic disorder, post-traumatic stress disorder, separation anxiety disorder, social anxiety disorder and specific phobia.

e Mild mood disorders include adjustment disorder with depressed mood, depressive disorder not otherwise specified, dysthymic disorder and mood disorder not otherwise specified.

f Manic disorders include bipolar disorder type I and type II, bipolar disorder not otherwise specified, cyclothymic disorder, hypomania and mania.

g Substance use disorders include alcohol abuse, alcohol dependence, alcohol use disorder not otherwise specified, substance abuse, substance dependence, substance use disorder not otherwise specified.

h Psychotropic medications include antidepressants, antipsychotics, methylphenidate and mood stabilizers.

Cohort-wide exclusion criteria were an IQ below 70, a major medical history or history of neurological illness, and for controls only, one or more first-degree relatives with a severe mood or psychotic disorder. Control offspring and their parents could have mild psychopathology to prevent having a “super-healthy” control group, which would unduly magnify potential group effects (Kendler et al., 2019). Participants were considered to be at familial risk if they had at least one first-degree or two second-degree relatives with BD or SZ. Given that the majority of the final sample comprises offspring (180 out of 187 participants; one was a sibling and six had two second-degree relatives) and for the sake of readability, we decided to use the term “offspring” for all participants. In the final sample, 8 BDo (from 5 families) had two parents with BD and 3 SZo (from 2 families) had one parent with SZ and one parent with BD; the rest of the high-familial-risk offspring had one parent with BD or SZ. Clinical diagnoses of index parents were confirmed using the Structured Clinical Interview for DSM-IV Axis I Disorders (SCID-I) (First and Gibbon, 2004). Parents of controls were screened for psychopathology with the MINI-Schedules for Clinical Assessment in Neuropsychiatry (Rehman, 2011), followed by a SCID-I in case of reported psychopathology.

At both waves, participants were psychiatrically evaluated using the Schedule for Affective Disorders and Schizophrenia for School-Age Children-Present and Lifetime Version (K-SADS-PL) (Kaufman et al., 2000) by interviewing participants and their parents separately. The summary information of this face-to-face semi-structured interview was used to establish the presence and severity of symptoms and current and lifetime DSM-IV axis I diagnoses. IQ was estimated using four subtests (block design, picture completion, information and vocabulary) of the Wechsler Intelligence Scale for Children-III (≤16 years) (Wechsler, 1991) or the Wechsler Adult Intelligence Scale-III (>16 years) (Wechsler Adult Intelligence Scale-Third Edition: Administration and scoring manual, n.d.). All assessments were conducted by trained interviewers with a bachelor’s or master’s degree in medicine or psychology.

Written informed consent was obtained from participants older than 12 years and from both parents or legal caregivers for participants aged between 8 and 18 years. Parents gave written consent for their own participation as well. The study was approved by the Medical Ethics Committee of the University Medical Center Utrecht and complies with the Declaration of Helsinki. Findings on (subsets of) the current study sample have been published before, but none of these included gyrification or sulcal morphometry (Collin et al., 2017, de Leeuw et al., 2017, Poortman et al., 2024a, Poortman et al., 2024b, Setiaman et al., 2023, van Haren et al., 2020).

### 2.2 Structural brain imaging

#### 2.2.1 MRI acquisition and preprocessing

MRI brain scans were obtained on a Philips 3 T Achieva or Philips 3 T Ingenia CX scanner (Philips Medical Systems, Best, the Netherlands) located at the University Medical Center Utrecht. See Supplement 1.2 for the distribution and comparison of how often each scanner was used per group and for acquisition parameters. We found no significant group discrepancies in scanner usage, except for a significant group difference between BDo and Co at wave 1 (*p* = 0.005).

FreeSurfer (v7.1.1) (Fischl, 2012) was used to process and segment the T1-weighted images. The Desikan-Killiany atlas was applied to parcellate the brain into 68 cortical regions of interest (ROIs) (34 per hemisphere) (Desikan et al., 2006). Visual quality control for volume, thickness and surface labeling was performed according to the ENIGMA procedures (http://enigma.ini.usc.edu/). Two researchers independently inspected the surface reconstruction and parcellation quality of each scan in the dataset. Additionally, as the Euler number is site-specific, scanner was centered by subtracting the scanner median of Euler number from all scans of each scanner (Kia et al., 2022, Rutherford et al., 2022). There were no group differences in scan quality. Supplement 1.2 provides additional information on preprocessing, and scan quality.

#### 2.2.2 Gyrification index and sulcal morphometry

For each ROI, the GI - defined as the ratio between the pial and hull surface areas - was calculated following the methodology of Schaer (Schaer et al., 2012). For each scan, the GI was calculated per vertex, and then averaged within each ROI to obtain the GI of each ROI. Additionally, total, hemispheric and lobar GI were calculated by weighting ROIs by their number of vertices and then averaging the GI values of all the ROIs belonging to the whole brain, a hemisphere or a lobe. A quality check was carried out to ensure the GI value exceeds 1 for each ROI, as a GI value lower than 1 would indicate a larger hull surface than the pial surface, which is not physiologically possible (Schaer et al., 2012). Sulcal measurements (i.e., sulcal depth, length and width) were obtained using the ENIGMA-Sulci protocol (Pizzagalli et al., 2020, Sun et al., 2022), which employs the morphologist pipeline from BrainVISA (v5.1.1), specifically the 'Import FreeSurfer grey/white segmentation to Morphologist' process (Borne et al., 2020). This pipeline imports the ribbon images from FreeSurfer and translates them into BrainVISA ontology to compute the sulcal measurements of 123 sulci (Perrot et al., 2011) (see the Supplement 1.3 for a detailed description, and STable S1 for the sulcal labels and descriptions). Total and hemispheric sulcal measurements were calculated as well by taking the average (for sulcal depth and width) or sum (for sulcal length) of the values of the respective measure of all sulci belonging to a hemisphere. The Desikan-Killiany regions used for the GI analyses and the sulci used for sulcal morphometric analyses are not completely distinct (i.e., a Desikan-Killiany region may include part of the sulcal bank and therefore overlap with a sulcus or sulci from the BrainVISA atlas). Thus, we consider these different analyses as complementary and do not aim to make direct comparisons between GI and the sulcal morphometric measures.

Not every sulcus could be identified in each scan (i.e., if sulcal depth, length and width were all missing), which meant that the corresponding sulcus was either absent in this subject or not measurable. Also, even if a sulcus was identified, for some sulci, depth but not length and width could be measured successfully. For example, when a region is buried deep in the brain, it may not touch the external brain hull, which is needed to measure sulcal length. In cases where sulcal length or width were not measured successfully, the software sets the values to zero. To prevent erroneously including them to our analyses, we treated these values as missing. We counted the number of identified and unidentified sulci, and we counted how often a measure (sulcal depth, length or width) was missing (see STable S1). The sulci that were most often not identifiable were typically minor sulci (e.g., the left and right diagonal ramus of the lateral fissure, anterior sub-central ramus of the lateral fissure and paracentral lobule central sulcus), and the main sulci for which certain measures were frequently missing were the left and right insula and posterior sub-central ramus of the lateral fissure (sulcal length in all four cases). This is highly consistent with previous studies (Pizzagalli et al., 2020, Sighinolfi et al., 2024). We used two cut-offs for the inclusion of a sulcus to each analysis, depending on the research question (see below).

### 2.3 Statistical analysis

#### 2.3.1 Demographic and clinical characteristics

Group differences in demographic and clinical characteristics were evaluated at both waves using Fisher’s exact test for categorical variables (i.e., sex, prevalence of lifetime psychiatric diagnosis, psychotropic medication use and scanner) and analyses of variance for continuous variables (i.e., age at scan, IQ, scan interval and summed present symptom scores of depression, mania and psychosis) followed by Tukey’s test in case of significant group effects.

#### 2.3.2 Age trajectories of gyrification and sulcal morphometry

To evaluate group differences in age trajectories of gyrification (lobar and regional) and the sulcal (hemispheric and regional) measures, a generalized additive mixed model (GAMM) analysis was performed using the *mgcv* package (v1.9–1) in R (v4.3.2) (R Core Team, 2022). GAMMs are used to enable the modeling of non-linear relationships between the dependent and predictor variables by adding smooth functions of the covariates (Wood, 2006). As with linear mixed-effects models, GAMMs make use of both the cross-sectional and longitudinal data in a dataset, such that no data has to be excluded, reducing bias and improving power. We used a factor-smooth approach that allowed comparison of the interactions between age at scan and group using the group variable (coded as ordered) in the *by* argument of the smooth term for age. A separate smooth term for age was added to represent the smooth effect of age in the reference level of the ordered group variable (once with controls as the reference level for the comparisons of each of the high-familial risk groups versus controls, and once with BDo as the reference level for the comparison between SZo and BDo) (Díaz-Caneja et al., 2021, Janssen et al., 2022, Sørensen et al., 2021). In GAMMs, the estimated degrees of freedom (*edf*) is an indication of the complexity or smoothness of the fitted term, with an *edf* (close to) 1 suggesting a (near-)linear effect, while a higher edf reflects a more flexible, non-linear effect. Group, sex, scanner and Euler number (FreeSurfer-generated proxy of scan quality (Rosen et al., 2018)) were added as fixed effects. Subject-ID and family-ID were included as random effects to account for within-subject and within-family effects, respectively. Based on previous literature, including studies on gyral and sulcal metrics (Bethlehem et al., 2022, Díaz-Caneja et al., 2021, Janssen et al., 2022, Mills et al., 2016) and based on visual inspection of the raw data, the level *k* was set at 4 for all analyses, using thin plate regression splines. We used the ‘k.check’ function from the *mgcv* package on the models on the lobar and hemispheric outcome measures to determine whether the chosen *k* level was adequate, and a higher *k* was not required in any of the models. To be included in the main analysis, comparing age-related differences between offspring groups of sulcal depth, length and width, a sulcus must have been identified in at least 75 % of scans (Dojat et al., 2018, Sun et al., 2022), and the respective measure should be successfully measured in at least 75 % scans as well (this was the case for all sulcal measures except for the sulcal length of the insula and posterior sub-central ramus of the lateral fissure) (STable S3). The formula of the regression is as follows: ‘gamm(BrainMeasure ∼ Group + Sex + Scanner + Euler + s(Age, *k* = 4, bs = “tp”) + s(Age, by = Group, *k* = 4, bs = “tp”), random = list(FamilyID = ∼1, SubjectID = ∼1), method= ”REML”)’. To examine mean group differences independent of age, the group variable from the GAMM models described above was evaluated.

Given that most longitudinal studies on brain development in high-familial-risk populations for SZ or BD thus far have used linear statistical models, we repeated our analyses using linear mixed model analyses for a closer comparison with these studies, using *lme4* (v1.1–35.5). This also provides betas of the age effects, allowing easier interpretation of the direction of the trajectories. For these analyses, age was centered around the grand mean for interpretation of the group comparisons (this centering was not needed for GAMM models as smooths are centered). To model the linear relationship between the brain measures and the predictors, this model included group, age, the interaction between group and age, sex, scanner and Euler number as fixed effects and subject ID and family ID as random effects, resulting in the following formula: ‘lmer(BrainMeasure ∼ Group + Age + Group *x* Age + Sex + Scanner + Euler + (1|FamilyID/SubjectID))’. Robust Effect Size Index (RESI) estimates were computed from the linear mixed-effects models (Vandekar et al., 2020).

#### 2.3.3 Sensitivity analyses

To explore the effects of IQ or symptom severity on our findings, the main analyses on total and lobar gyrification and total hemispheric sulcal morphometric measures were repeated once with IQ and once with the K-SADS summed scores of depression, mania and psychosis (three separate variables) added as fixed effects.

#### 2.3.4 Sulci identification

To determine whether there were group differences in whether a sulcus was successfully identified or not, a generalized linear mixed-effects model was applied to 37 sulci that were identified in less than 90 % of the scans (marked yellow or blue in STable S1). The following formula was used: ‘glmer(SulcusIdentifiedYorN ∼ Group + Scanner + Euler + (1|FamilyID/SubjectID), family = binomial)’.

#### 2.3.5 Multiple comparison correction

To correct for multiple comparisons, we applied a Benjamini-Hochberg False Discovery Rate (FDR) correction (*q*=0.05) (Benjamini and Hochberg, 1995) once on age in controls, once on the pairwise group comparisons, and once on the smoothed age*group comparisons, using the *p.adjust* function from the R package *stats*. For the global analyses, FDR correction was applied on all lobes for gyrification (2 ×5 =10 ROIs in total), and on the hemispheres for all three sulcal measures (3 ×2 =6 ROIs in total). In the regional analyses, FDR correction was applied on all 68 ROIs for the gyrification analyses, and on the 110 ROIs for each of the analyses on the three sulcal measures (123 sulci minus the 13 sulci that were excluded due to being identified in less than 75 % of scans). In the sulcus identification analyses, FDR correction was applied on the 37 ROIs.

## 3 Results

### 3.1 Demographic and clinical characteristics

Table 1 provides an overview of the demographic and clinical characteristics of each group. Since there were group differences in age, sex, IQ and psychopathology, we included these as control variables (age and sex in all models; IQ and psychopathology in sensitivity analyses). See Supplement 1.1 for the attrition bias analyses.

### 3.2 Age trajectories of gyrification and sulcal morphometry in controls

110 out of 123 sulci were included in the trajectory analyses as they were identified in at least 75 % of the scans (STable S1).

**Fig. 2:**
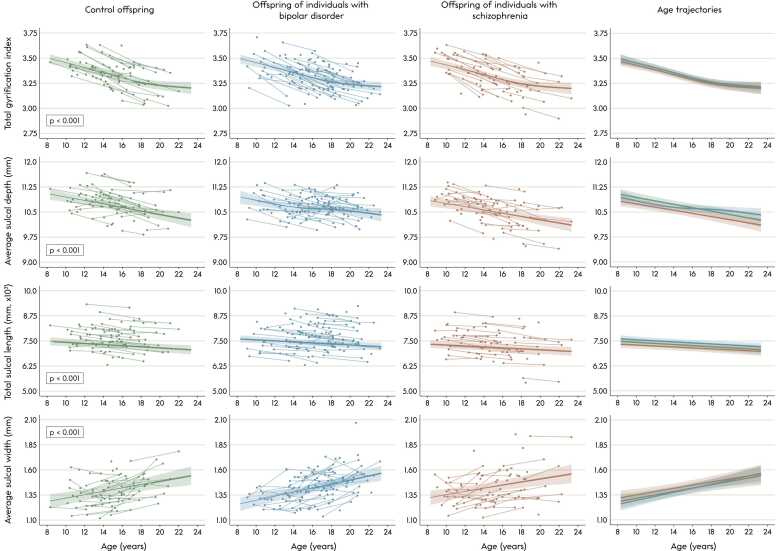
Age trajectories (in years) of total brain gyrification index, average sulcal depth, total sulcal length and average sulcal width. Generalized additive mixed model (*k* = 4) fits and 2-standard-error bands are presented on top of the raw data. The gyrification index, average sulcal depth and total sulcal length decreased and average sulcal width increased significantly with age in controls (all p’s < 0.001). After multiple comparison correction, there were no significant differences in the age trajectories between the three groups. Note that the fits may be slightly vertically shifted compared to the raw data points due to age being centered in the generalized additive mixed model, as this influences the intercept some.

**Fig. 3:**
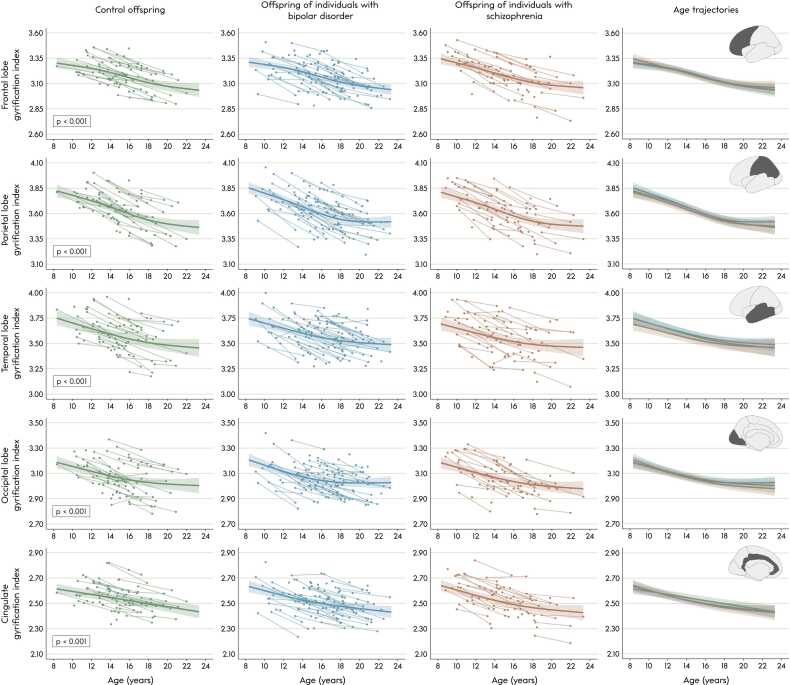
Age trajectories (in years) of gyrification of the bilateral frontal, parietal, temporal and occipital lobes and cingulate cortex. Generalized additive mixed model (*k* = 4) fits and 2-standard-error bands are presented on top of the raw data. There was a significant effect of age in controls in all bilateral lobes (all *p*’s ≤ 0.001), where the gyrification index decreased with age in all lobes. After multiple comparison correction, there were no significant differences in age trajectories between the three groups. Note that the fits may be slightly vertically shifted compared to the raw data points due to age being centered in the generalized additive mixed model, as this influences the intercept some. The highlighted lobes in the right-most figures are presented on the left hemisphere purely for visualization purposes.

In controls, there were significant age effects in gyrification of all lobes (*p*’s < 0.001), and significant age effects in average sulcal depth, total sulcal length and average sulcal width of the left and right hemispheres (*p*’s ≤ 0.015). Gyrification decreased (near-)linearly with age in all left and right lobes (edf’s between 1.000 and 2.843). Left and right hemispheric average sulcal depth and total sulcal length decreased linearly (edf’s between 1.000 and 1.003), and average sulcal width increased with age (edf’s of 1.823 and 1.000 for left and right hemisphere, respectively). See Fig. 2, Fig. 3 for GAMM trajectories of bilateral measures, and see STables S2–S5 for GAMM output.

Regionally, widespread significant age effects in gyrification were found in 94 % of the regions in controls (31 and 33 in the left and right hemispheres, respectively, out of the total 68 ROIs. For sulcal depth, significant age effects were observed in respectively 5 % of the sulci (3 in each hemisphere, out of 110 sulci) and for sulcal width in 31 % of the sulci (19 and 15 in the left and right hemisphere, respectively), which were mostly (near-)linear as well. In local sulcal length, no significant age effects were found.

**Fig. 4:**
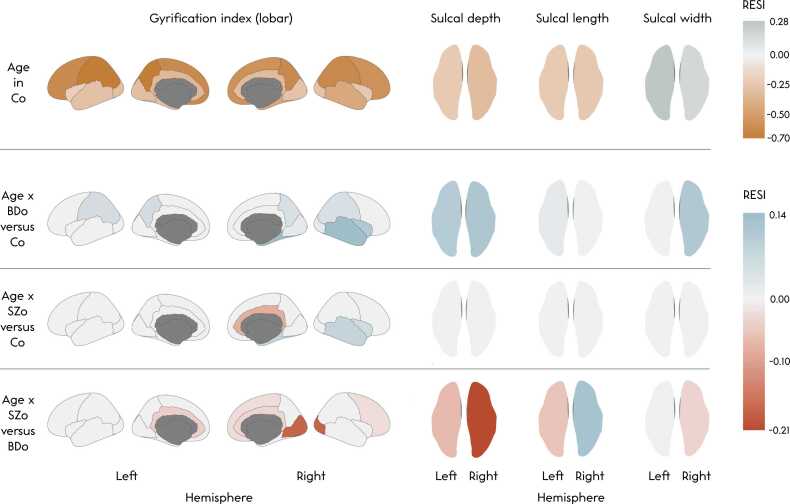
Brain color maps of Robust Effect Size Index (RESI) estimates for the effect of age in controls and of each age*group pairwise comparison in the linear mixed-effects model analyses on lobar gyrification and hemispheric average sulcal depth, total sulcal length and average sulcal width. Note that while the RESI itself is an index, a positive or negative sign is assigned based on the direction of the effect. For the effect sizes of the group comparisons, red reflects regions where the first group has a lower increase or steeper decrease than the second group, and blue-grey reflects regions where the first group has a higher increase or less steep decrease than the second group.

Linear mixed-effects model analyses yielded highly similar findings in gyrification patterns, with a decrease in gyrification of all lobes (*p*’s < 0.001), a decrease in left and right hemispheric sulcal depth and length, and an increase in left and right hemispheric sulcal width with increasing age (*p*’s ≤ 0.015). Regionally, a decrease in gyrification of the same regions (94 %) as in the GAMM analyses was found. For sulcal depth, significant age effects were observed in 2 % (1 sulcus in each hemisphere) and in sulcal width, in 28 % (18 and 13 in the left and right hemisphere, respectively). For sulcal length, no significant age effects were found in the sulci. See SFigures S2 and S3 for linear mixed-effects model trajectories of bilateral measures, and see STables S6–S9 for linear mixed-effects model output. RESI estimates ranged between −0.70 and 0.28 for the age effect in controls (Fig. 4).

For a distribution of sex in each of the three groups for the four global measures, see Supplemental Figure S4.

### 3.3 Group differences in age trajectories of gyrification and sulcal morphometry

The GAMM analyses revealed no significant group differences in age trajectories of total or lobar gyrification or of total or hemispheric sulcal depth, length or width (Fig. 2, Fig. 3; STables S2–S5). For completeness, the trajectories of global average sulcal depth and width weighted by the length or surface of the sulci are also provided, which showed similar patterns (SFigure S5).

Regionally, GAMM analyses showed no significant group differences in age trajectories between groups in GI or sulcal depth, length or width.

There were no significant main effects of the group variable on global or regional GI, or sulcal morphometric measures (correcting for age and age*group interactions).

**Fig. 5:**
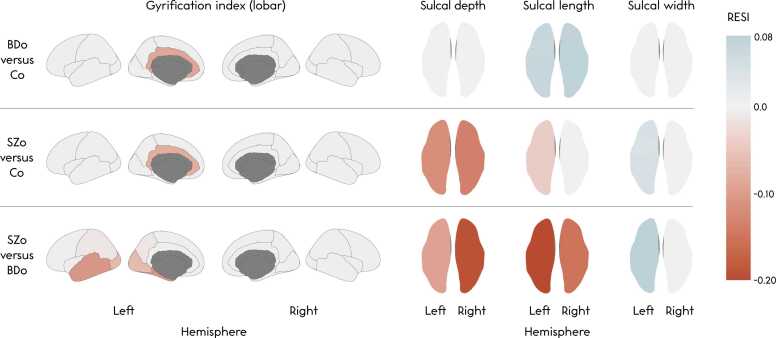
Brain color maps of Robust Effect Size Index (RESI) estimates for each pairwise group comparison (the group variable) in the linear mixed-effects model analyses on lobar gyrification and hemispheric average sulcal depth, total sulcal length and average sulcal width. Note that while the RESI itself is an index, a positive or negative sign is assigned based on the direction of the effect. Red reflects regions where the first group has lower values than the second group, and blue-grey reflects regions where the first group has higher values than the second group.

Linear mixed-effects model analyses also did not reveal significant mean group differences or group differences in age trajectories of lobar or regional gyrification, or hemispheric or regional sulcal depth, length or width (SFigures S2 and S3; STables S6–S9). Fig. 4, Fig. 5 summarize the RESI estimates on brain color maps for the age*group and the group effects from the linear mixed-effects model analyses, respectively. The RESI estimates ranged between −0.21 and 0.14 for the age*group interactions and between −0.20 and 0.08 for the mean group effects.

### 3.4 Sensitivity analyses

Repeating the analyses with IQ or summed symptom scores of depression, mania and psychosis added as fixed effects did not change the results in any of the analyses (STables S10 and S11).

### 3.5 Sulci identification

37 out of the original 123 sulci were identified in less than 90 % of scans and thus included in the sulci identification analyses (STable S1). In these sulci, we investigated whether groups differed in how likely a sulcus was identified or not. In the individuals with two scans, the rate of sulcus identification consistency across the two scans was high (≥0.80) (STable S12). Generalized linear mixed-effects models showed no significant group differences in the likelihood of a sulcus being identified (all *p*’s ≥ 0.007, STable S13).

## 4 Discussion

In this prospective, longitudinal, cross-disorder study, we investigated the developmental trajectories of gyrification and sulcal measurements (i.e., depth, length and width) in child and adolescent offspring at increased familial risk for severe mental illness. As expected, gyrification, sulcal depth and sulcal length decreased and sulcal width increased with age in controls in a (near-)linear fashion, congruent with the recurrent finding of a flattening cortex during adolescence (Alemán-Gómez et al., 2013, White et al., 2010). Globally, sulcal length decreased marginally with age as well. Regarding the first research question, we did not find differences in age trajectories of gyrification or overall mean gyrification between SZo, BDo and controls. Similarly, with respect to our second hypothesis, we found no significant group differences in the age trajectories or overall mean values of sulcal depth, length or width. Finally, there was no difference between groups in the likelihood of a sulcus being identified. Controlling for IQ or the summed symptom scores of depression, mania and psychosis did not significantly alter the findings of the main analyses. Our findings suggest that high familial risk for BD or SZ is not associated with deviations in trajectories of cortical folding characteristics during childhood and adolescence.

Developmental trajectories of gyrification and sulcal morphometrics were highly similar between the high-familial-risk groups and controls, showing predominantly (near-)linear trends with age in all three groups. These age trajectories are in line with previous studies in general population samples (Klein et al., 2014, White et al., 2010). Regarding SZo, the finding of preserved trajectories is incongruous with earlier research, which generally showed particularly frontal hypergyrification in both individuals with SZ and those at familial and clinical high-risk risk during late adolescence and young adulthood (Sasabayashi et al., 2021). However, the studies included in the review were cross-sectional and compared means across individuals representing a wide age range, complicating the comparison with our study findings. One recent longitudinal study found a progressive decline of gyrification of fronto-temporal regions in young adult individuals with schizophrenia compared to controls, but not in those with schizotypal disorder (which is related to higher clinical risk to develop SZ, Barrantes-Vidal et al., 2020). However, the sample size of this study was small (Pham et al., 2021). To capture trajectories of brain morphometry accurately and reliably, longitudinal data of large sample sizes are required, preferably with more than two assessments per individual (King et al., 2018, Parsons and McCormick, 2022). Moreover, our dataset had limited data points at the front and tail ends. Following up the participants in our study is therefore essential. This will also result in more data points past the typical age of illness onset, potentially allowing us to stratify the high-risk offspring into those who develop a diagnosis and those who do not.

Controlling for IQ or psychopathology in our sensitivity analyses did not change the main findings. This corroborates our earlier work on the association between familial risk for severe mental disorder and developmental trajectories of cortical and subcortical brain structure and structural network features in the same cohort (Poortman et al., 2024b, Poortman et al., 2024a). Our sensitivity analyses did, however, show that IQ associated positively with sulcal depth, which corresponds with previous research where higher IQ was found to be linked to higher fractal dimension and higher frequency of sulcal pits (Im et al., 2011, Im et al., 2006). Furthermore, we found depressive symptoms to be negatively associated and psychotic symptoms positively associated with GI, which is in accordance with studies that found decreased gyrification in major depression (Han et al., 2025, Kang et al., 2023, Natsuyama et al., 2022, Shen et al., 2024, Zhang et al., 2009) and hypergyrification in psychosis (Basavaraju et al., 2023, Sasabayashi et al., 2020, Sasabayashi et al., 2017, Zuliani et al., 2018) (but see (Drobinin et al., 2020)). These findings provide suggestive evidence for IQ or symptom severity to explain some of the variance in gyrification and sulcal morphometry changes over time. Larger samples including more within-subject data are needed to verify this claim and to provide information on the causality of such effects.

The gyral and sulcal patterns of the brain are often considered as a neurodevelopmental marker. The most pronounced folding changes take place before the second year of age, with high regional heterogeneity in gyral growth rate (Li et al., 2014). After this initial period of rapid evolvement, global sulcal patterns have been shown to remain relatively stable throughout the lifespan (Cachia et al., 2016, de Vareilles et al., 2023), with only subtle modifications during childhood (Blanton et al., 2001) and a flattening cortex during adolescence (Alemán-Gómez et al., 2013). Together with increasing evidence that schizophrenia may be the result of aberrant brain development in pre-, peri- and neonatal stages (Birnbaum and Weinberger, 2024, Ursini et al., 2021), it is not unlikely that sulcal abnormalities already emerge during this stage. This notion is supported by the lower sulcal depth, on average, seen in the SZo group across the entire age range, which, although it did not survive the multiple comparison correction, is widespread across the brain (STables S3 and S7). Alternatively, it is possible that anomalous gyrification and sulcal development occur in high-familial-risk offspring in the first years after birth, but trajectories converge towards that of control offspring again as they grow older, an idea that has been postulated recently as well, although these trajectories are putative and require confirmation (Sasabayashi et al., 2021). Future neuroimaging studies on brain folding in offspring at high familial risk for schizophrenia or bipolar disorder should ideally include, in addition to larger samples and longer follow-ups, assessments in the fetal, neonatal and infancy period.

One of the factors that make it difficult to establish neural markers of high familial risk for SZ or BD in children and adolescents is the high heterogeneity within these groups. Individuals in our sample varied in diagnostic status (e.g., developmental disorders, anxiety disorders, (mild) mood disorders, no diagnosis) (Poortman et al., 2024a, Setiaman et al., 2023) and in genetic load, parenting environment and intrauterine environment. These genetic and environmental factors can also affect the process of gyrification and are, by themselves and in interaction, an explanation for why brain abnormalities are found in patient populations (Kelly et al., 2016, Quezada et al., 2018). It would therefore be important to examine to what extent abnormalities in gyrification or sulcal morphometrics in schizophrenia or bipolar disorder are explained by genetic and environmental risk factors for these disorders.

The current study is one of the few studies on GI and sulcal morphometry in child and adolescent individuals at high familial risk for SZ and BD and unique in its consideration of (potentially non-linear) longitudinal trajectories. Several limitations should nonetheless be acknowledged when interpreting the findings of this study. First, the relatively modest sample size and resulting statistical power may have prevented us from picking up subtle differences in the trajectories of GI or sulcal measurements. Similar to other structural brain measures (de Zwarte et al., 2019, Poortman et al., 2024b), effect sizes of group differences in gyrification and sulcal measures were low. Moreover, ages at the extremes (≤10 years and ≥22 years) contain fewer scans leading to an underrepresentation in all three groups. Therefore, follow-up at older ages is required for more robust modeling of the trajectories around early childhood and late adolescence. Second, related to the first point, our sample size also did not enable us to further investigate sex-related effects as it does not render sufficient statistical power for a three-way interaction of sex, age and group. Sex differences have been reported in the prevalence of psychiatric disorders (Merikangas et al., 2010), as well as in (the development of) sulcal measures and gyrification during adolescence (Mutlu et al., 2013, Raznahan et al., 2011, Salminen et al., 2022, Torgerson et al., 2024). Future studies examining brain development in the psychiatric population should explore sex differences using larger samples. Third, different atlases were used for FreeSurfer’s local GI (Desikan et al., 2006) and BrainVISA’s sulcal morphometrics (Perrot et al., 2011), hindering the direct investigation of local associations between the measures. Fourth, while visual quality control was performed on the FreeSurfer surface reconstruction of each scan and we controlled for Euler number in our analyses, the anatomical validity of the entire set of sulci was not explicitly evaluated for each individual MRI scan. Recent research has shown that certain sulci can sometimes be partially mislabelled by the BrainVISA algorithm (Sighinolfi et al., 2024) and, ideally, these sulci are manually relabelled for improved anatomical accuracy. Fifth, in-scanner head motion has been shown to cause systematical bias in estimates of brain morphometrical measures in pediatric neuroimaging (Alexander-Bloch et al., 2016, Pardoe et al., 2016, Rosen et al., 2018). To address this potential issue in our own data, we performed rigorous visual quality control and added the Euler number as a proxy of scan quality. Reassuringly, in our dataset, there were no group differences or interaction between group and age in the Euler number; consequently, we are confident that our findings are not explained by head movement. Sixth, data was acquired on two scanners due to a scanner upgrade mid-study, which may introduce bias and reliability issues (Medawar et al., 2021), although the brand, field strength and acquisition parameters were identical keeping differences as minimal as possible. Still, the scanner was added as a covariate in the analyses to correct for its effect on the variance. Importantly, there were no group differences or interaction between group and age in scanner use. Finally, it should be noted that the majority of the offspring in this study have not yet reached the typical age of onset at of BD and SZ (Dalsgaard et al., 2020, Kessler et al., 2005, Solmi et al., 2022). Over time, more high-familial-risk offspring are likely to develop psychotic or mood symptoms or disorders (Mesman et al., 2013, Rasic et al., 2014, Uher et al., 2023), which will allow us to investigate resilience by stratifying the high-familial-risk offspring groups by disorder manifestation.

In conclusion, our study shows decreasing age trajectories of gyrification, sulcal depth and, to a lesser extent, sulcal length, and increasing sulcal width with age, chiefly linear in shape. These trajectories did not differ between SZo, BDo and controls, suggesting relatively preserved development of brain folding patterns during childhood and adolescence in individuals at high familial risk for major psychiatric disorder. Prospective follow-up research in these offspring beyond the usual age of onset of mood and psychosis-spectrum disorders is imperative. This will not only help to understand how brain development pertains to high familial risk for mental illness but also allow us to determine whether developmental trajectories differ between those who go on to develop severe mental illness themselves, which could aid in early prediction, and those who remain relatively unaffected despite their increased familial risk, potentially revealing resilience factors that may ultimately benefit tailored intervention.

## Funding

This work was supported by the 10.13039/100000874Brain and Behavior Research Foundation (2013–2015 10.13039/100009670NARSAD Independent Investigator Grant No. 20244 [to MHJH]), The Netherlands Organization for Scientific Research (2012–2017 VIDI Grant No. 452–11–014 [to NEMvH]), the Sophia Foundation (WAR20–40 [to NEMvH]) and the 10.13039/501100000780European Union’s HorizonEurope Research and Innovation Program (FAMILY; Grant No. 101057529 [to NEMvH]). There was no involvement by the funding bodies at any stage of the study.

## CRediT authorship contribution statement

**Barendse Marjolein E.A.:** Writing – review & editing, Validation, Supervision, Methodology, Formal analysis, Conceptualization. **van Haren Neeltje E.M.:** Writing – review & editing, Supervision, Resources, Project administration, Methodology, Investigation, Funding acquisition, Formal analysis, Conceptualization. **Poortman Simon R.:** Writing – review & editing, Writing – original draft, Visualization, Validation, Software, Methodology, Formal analysis, Data curation, Conceptualization. **Jamarík Jakub:** Writing – review & editing, Validation, Software, Methodology, Formal analysis. **ten Harmsen van der Beek Louise:** Writing – review & editing, Writing – original draft, Software, Formal analysis, Data curation, Conceptualization. **Setiaman Nikita:** Writing – review & editing, Project administration, Investigation, Data curation. **Hillegers Manon H.J.:** Writing – review & editing, Resources, Project administration, Funding acquisition, Conceptualization.

## Declaration of Competing Interest

The authors declare that they have no known competing financial interests or personal relationships that could have appeared to influence the work reported in this paper.

## Data Availability

Data will be made available on request.
