# Supplementary material for: Developmental trajectories of gyrification and sulcal morphometrics in children and adolescents at high familial risk for bipolar disorder or schizophrenia
Source: Dev Cogn Neurosci. 2025 Feb 24;72:101536. doi: 10.1016/j.dcn.2025.101536 (PMC11919454; doi:10.1016/j.dcn.2025.101536)
Supplement: Supplementary file 1 — Supplementary material [file mmc1.docx]

**Developmental trajectories of gyrification and sulcal morphometrics in children and adolescents at high familial risk for bipolar disorder or schizophrenia**

***Supplemental Information***

*1.1 Scan exclusion overview and attrition bias analyses*

Exclusion reasons per group per wave

|  | Wave 1 | | | |  | Wave 2 | | | |
| --- | --- | --- | --- | --- | --- | --- | --- | --- | --- |
|  | BDo | SZo | Co | Total |  | BDo | SZo | Co | Total |
| N original | 102 | 66 | 64 | 232 |  | 102 | 66 | 64 | 232 |
| Incidental finding (e.g., cyst) | 4 | 1 | 0 | 5 |  | 4 | 1 | 0 | 5 |
| 1x SDR | 0 | 3 | 2 | 5 |  | 0 | 3 | 2 | 5 |
| 1x sibling | 1 | 0 | 0 | 1 |  | 0 | 0 | 0 | 0 |
| IQ below 70 | 1 | 0 | 0 | 1 |  | 1 | 0 | 0 | 1 |
| Twin(s) | 4 | 1 | 0 | 5 |  | 0 | 0 | 0 | 0 |
| Exclusion criteria Co^a^ | 0 | 0 | 5 | 5 |  | 0 | 0 | 5 | 5 |
| MRI incompatible^b^ | 10 | 6 | 0 | 16 |  | 6 | 3 | 2 | 11 |
| Poor scan quality | 14 | 1 | 12 | 27 |  | 1 | 3 | 1 | 5 |
| Other^c^ | 14 | 12 | 1 | 27 |  | 5 | 7 | 2 | 14 |
| Dropout | 0 | 0 | 0 | 0 |  | 13 | 6 | 7 | 26 |
| N total excluded | 48 | 24 | 20 | 92 |  | 30 | 23 | 19 | 72 |
| N after exclusion | 54 | 42 | 44 | 140 |  | 72 | 43 | 45 | 160 |

BDo = offspring of individuals with bipolar disorder, Co = control offspring, SDR = second-degree relative, SZo = offspring of individuals with schizophrenia

^a^One or more first-degree relatives of five control offspring had psychopathology.

^b^Reasons include braces, piercings or metal in the body.

^c^During wave 1, children and adolescents who were left-handed were excluded as at the time this was common practice for task-based fMRI studies. During wave 2, these were no longer exclusion criteria and participants were invited for an MRI.

Attrition bias analyses were performed to compare excluded participants (those without available MRI data) with included participants (those with available MRI data on at least one wave) on their demographic and clinical characteristics at inclusion (wave 1), to determine whether scan exclusion was driven by age, group, sex, psychotropic medication use, or symptom scores of depression, mania or psychosis. For each of these variables, a generalized linear model was run as follows: ‘glm(AnyUsableDataYorN ~ DemographicOrClinicalVariable, family = binomial)’. This did not reveal any significant differences for age (*p*=0.270), group (*p*=0.632), sex (*p*=0.373), psychotropic medication use (*p*=0.988) or the summed symptom scores of depression (*p*=0.312) and mania (*p*=0.436). There was a significant effect of the summed symptom score of psychosis (*p*=0.023), such that participants with more psychotic symptoms at wave 1 were less likely to have (good quality) MRI data.

The proportion of total scan exclusions did not differ significantly between the three groups (*p*=0.354).

*1.2 Neuroimaging*

*Scanner group comparisons*

With respect to the number of offspring scanned with each scanner (Achieva or Ingenia CX), a significant group difference was found at wave 1 (*p*=0.014), with pairwise comparisons showing a significant difference between offspring of individuals with bipolar disorder (BDo) and control offspring (Co) (*p*=0.005). Therefore, we controlled for scanner in all models. No significant group differences in number of offspring scanned with each scanner were found at wave 2 (*p*=0.954), nor between offspring who switched scanners and those who did not (*p*=0.083).

Number of offspring per group scanner with each scanner at each visit

|  | Wave 1 | | Wave 2 | | Scanner change^a^ | No scanner change | |
| --- | --- | --- | --- | --- | --- | --- | --- |
|  | Achieva | Ingenia CX | Achieva | Ingenia CX |  | Achieva | Ingenia CX |
| BDo | 52 | 2 | 25 | 47 | 31 (67%) | 13 | 2 |
| SZo | 36 | 6 | 14 | 29 | 19 (59%) | 10 | 3 |
| Co | 34 | 10 | 16 | 29 | 15 (43%) | 11 | 9 |
| Total | 122 | 18 | 55 | 105 | 65 | 34 | 14 |

BDo = offspring of individuals with bipolar disorder, Co = controls, SZo = offspring of individuals with schizophrenia

^a^These numbers concern the 46 BDo, 32 SZo and 35 Co that were scanned at both waves.

Considering the entire sample (N=300), to determine whether there are group differences in how many times each of the two scanners was used, and whether the effect of age on which scanner was used differs between groups, we a ran a generalized linear model as follows: ‘Scanner ~ Group + Age + Group x Age’, which showed that there are no significant (age-related) group differences in which of the two scanners was used (all *p*’s>0.102).

Generalized linear model (Scanner ~ Group + Age + Group x Age)

| Scanner (Achieva or Ingenia CX) | *ß* | Standard error | *z* | *p* |
| --- | --- | --- | --- | --- |
| (Intercept)^a^ | -5.365 | 1.465 | -3.663 | <0.001 |
| BDo vs Co^a^ | -0.434 | 1.954 | -0.222 | 0.824 |
| SZo vs Co^a^ | 2.355 | 1.837 | 1.282 | 0.200 |
| SZo vs BDo^b^ | 2.789 | 1.703 | 1.637 | 0.102 |
| Age in Co^a^ | 0.337 | 0.095 | 3.555 | **<0.001** |
| Age x BDo vs Co^a^ | -0.015 | 0.121 | -0.125 | 0.900 |
| Age x SZo vs Co^a^ | -0.161 | 0.119 | -1.360 | 0.174 |
| Age x SZo vs BDo^b^ | -0.146 | 0.104 | -1.406 | 0.160 |

BDo = offspring of individuals with bipolar disorder, Co = controls, SZo = offspring of individuals with schizophrenia

^a^Analyses run with control offspring as reference group.

^b^Analyses run with offspring of individuals with bipolar disorder as reference group.

*MRI acquisition parameters*

## Three-dimensional T1-weighted sagittal spoiled-gradient fast-field echo scans of the whole brain were acquired for tissue classification and cortical parcellation, with the following parameters for both scanners: 200 contiguous slices; 0.75x0.75x0.80 mm3 voxel size, TE=4.6 ms, TR=10 ms, flip angle=8°, field of view (FOV)=240x240x160 mm3, eight-channel head coil, acquisition time=10 minutes.

##

## *MRI preprocessing*

## All imaging data were coded to ensure blinding for participant identification and diagnoses during image processing. Imaging data were (pre)processed and analysed on SURFsara’s Snellius compute cluster. Before migration to the compute cluster, T1-weighted images were defaced using mri_deface version 1.22 (Bischoff-Grethe et al., 2007). Visual quality control was performed by S.P. to determine whether defacing was performed successfully (i.e., the participant’s face was made unrecognisable with the brain left intact). FreeSurfer (v7.1.1) (Fischl, 2012) was used to process and segment the T1-weighted images. T1-weighted image processing included automated Talairach transformation, intensity normalisation, removal of non-brain tissue, segmentation of subcortical white matter and grey matter, tessellation of the grey/white matter boundary, and automated topology correction (Dale et al., 1999; Fischl et al., 2002, 1999; Rosas et al., 2002).

*Scan quality*

In addition to rigorous visual quality control of the T1-weighted scans and correction for scan quality in our models, we investigated potential group differences in scan quality statistically. For each T1-weighted scan, we calculated the Euler number for each hemisphere using the surface holes given by FreeSurfer’s recon-all. To see whether there was an effect of age and whether this differed per group, we ran an ANOVA for each hemisphere on the scanner-median-centered Euler number as follows: ‘Euler ~ Group + Age + Group x Age’. There were no significant group differences (Group: *p*=0.919 and *p*=0.297 for left and right hemisphere, respectively) and there was an effect of age (Age: *p*=.003 and *p*=0.012 for left and right hemisphere, respectively), but this did not differ significantly between groups (Group x Age: *p*=0.870 and *p*=0.551 for left and right hemisphere, respectively).

Unsurprisingly, Euler number had a significant effect in a considerable number of the models even after scans of poor quality were excluded, in line with earlier research (Rosen et al., 2018), although betas are marginal. Typically, younger individuals are more prone to moving in the scanner (Pardoe et al., 2016), and head motion has been shown to remain correlated with age even after stringent quality control in child and adolescent samples (Baum et al., 2018; Satterthwaite et al., 2012). Thus, given the study’s age range, the Euler number is directly associated with age, and age is expected to have strong effects in structural (including gyral) brain development during childhood and adolescence (Mills et al., 2016; White et al., 2010).

*1.3 Sulcal measurements*

BrainVISA creates a grey matter/cerebrospinal fluid (GM/CSF) mask from the ribbon image, allowing for the automatic segmentation of cortical sulci throughout the cortex. This segmentation corresponds to the crevasses at the base of the “landscape”, with the altitude defined by image intensity. This methodology provides a robust sulcal surface definition that remains unaffected by variations in cortical thickness or width, or grey matter/white matter contrast (Jouvent et al., 2011; Ochiai et al., 2004). BrainVISA automatically segments and labels 123 sulci (Perrot et al., 2011). Sulcal depth is calculated as the geodesic distance between the sulcal base and the hull, averaged across all points along the sulcus. Sulcal length is determined on the hull and defined as the length of the external line of the sulcus, the line which joins the fold to the brain hull. Sulcal width is calculated as the width of the CSF in the sulcus: it is the volume of CSF in the sulcus, divided by the surface of the skeleton mesh. All sulcal measurements (n = 369) are conducted in the Talairach space of the subject's images.


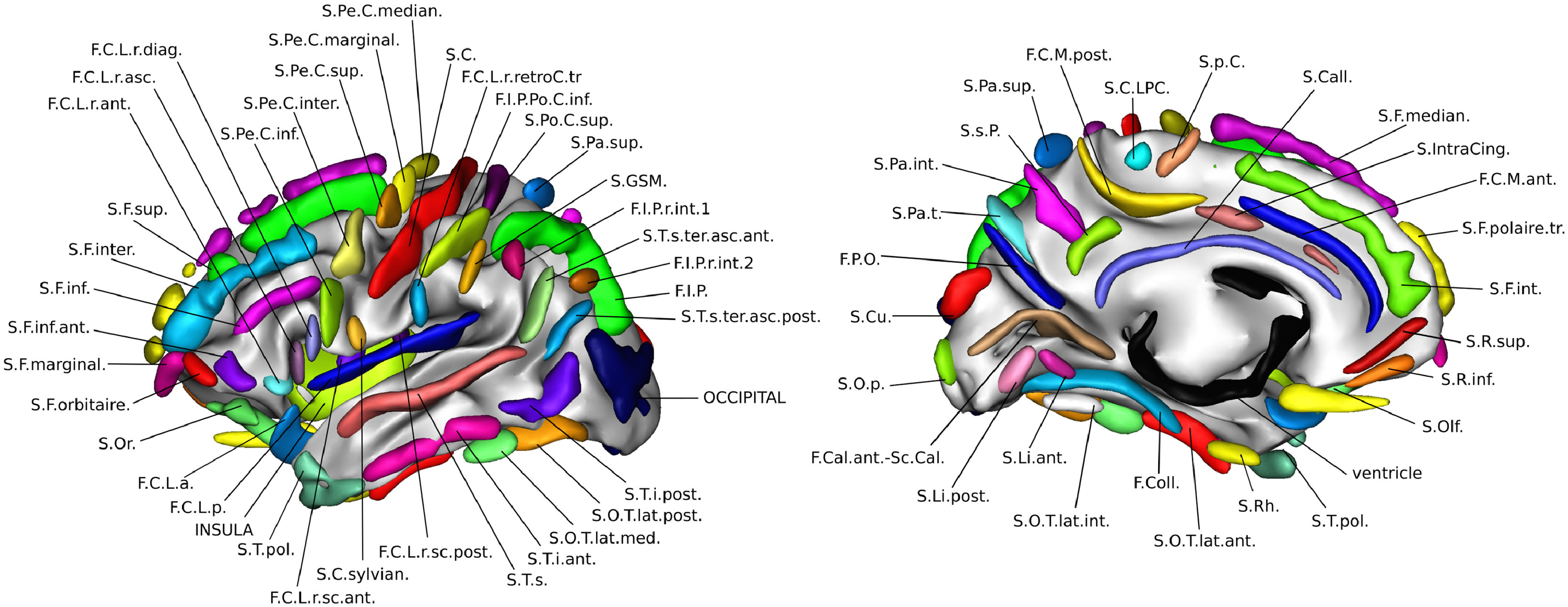


Sulcal labels used for the analyses of sulcal depth, length and width.


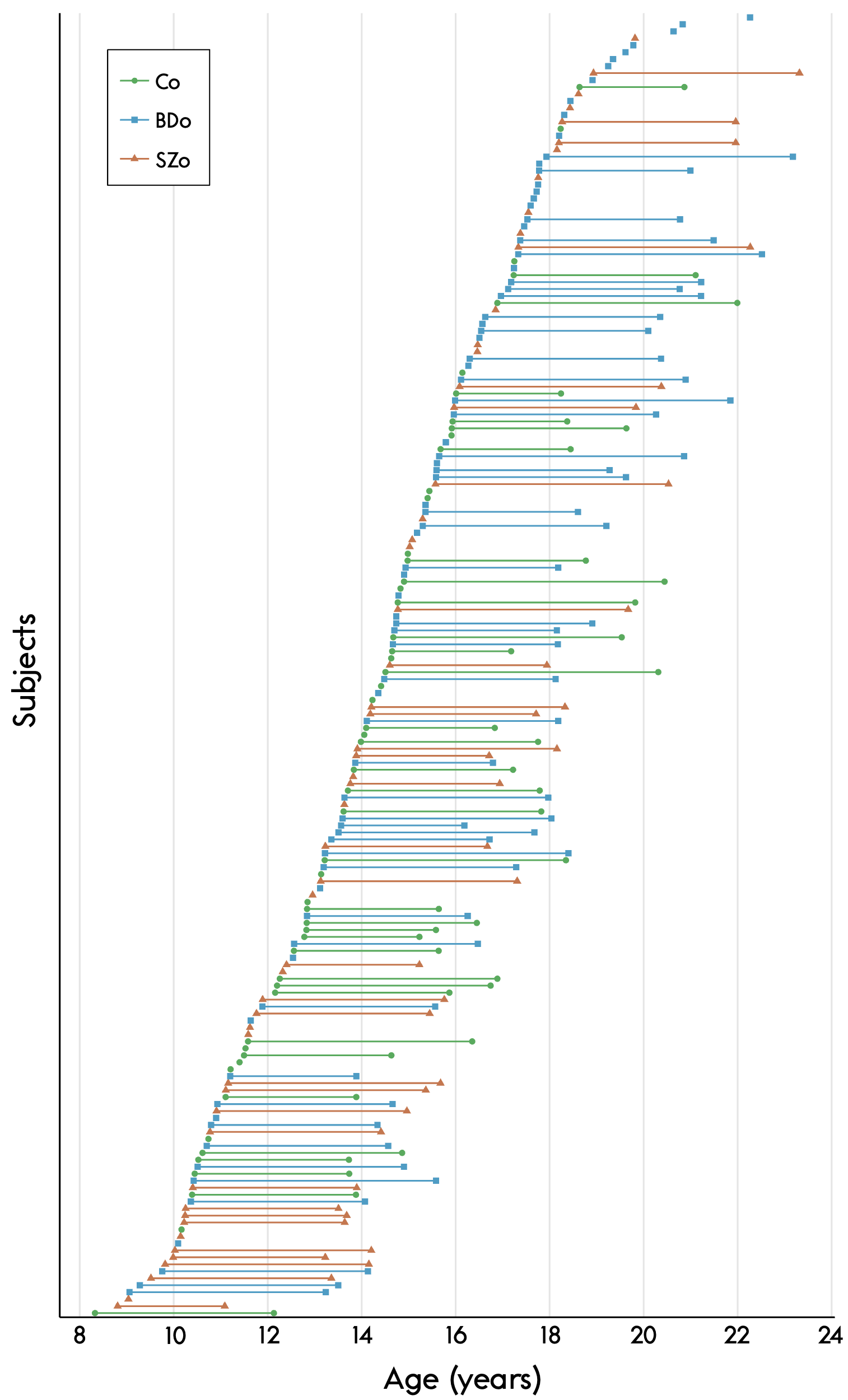


**Supplemental Figure S1.** Scatter plot of age at scan (in years) for all participants. Each participant is shown in a separate row, with each line connecting the two scans for the subjects that underwent a repeated assessment. BDo = bipolar disorder offspring, Co = control offspring, SZo = schizophrenia offspring

**Supplemental Figure S2.** Age trajectories (in years) of total brain gyrification index, average sulcal depth, total sulcal length and average sulcal width. Linear mixed-effects model fits and 2-standard-error bands are presented on top of the raw data. The gyrification index, average sulcal depth and total sulcal length decreased and average sulcal width increased significantly with age in controls (all *p*’s<0.001). After multiple comparison correction, there were no significant differences in the age trajectories between the three groups.

**Supplemental Figure S3.** Age trajectories (in years) of gyrification of the bilateral frontal, parietal, temporal and occipital lobes and cingulate cortex. Linear mixed-effects model fits and 2-standard-error bands are presented on top of the raw data. There was a significant effect of age in controls in all bilateral lobes (all *p*’s≤0.001), where the gyrification index decreased with age in all lobes. After multiple comparison correction, there were no significant differences in age trajectories between the three groups. The highlighted lobes in the right-most figures are presented on the left hemisphere purely for visualization purposes.

**Supplemental Figure S4.** Total gyrification, average sulcal depth, total sulcal length and average sulcal width as a function of age per group, with colors based on biological sex (orange = female; green = male). Raw data points are presented.

**Supplemental Figure S5.** Age trajectories (in years) global average sulcal depth and width, weighted by the length or surface of each sulcus. Generalized additive mixed model (k=4) fits and 2-standard-error bands are presented on top of the raw data. As with the main measures, the weighted average sulcal depth measures decreased and weighted average sulcal width measures increased with age in controls (all *p*’s<0.001). After multiple comparison correction, there were no significant differences in the age trajectories between the three groups.

**REFERENCES**

Baum, G.L., Roalf, D.R., Cook, P.A., Ciric, R., Rosen, A.F.G., Xia, C., Elliott, M.A., Ruparel, K., Verma, R., Tunç, B., Gur, R.C., Gur, R.E., Bassett, D.S., Satterthwaite, T.D., 2018. The impact of in-scanner head motion on structural connectivity derived from diffusion MRI. Neuroimage 173, 275–286.

Bischoff-Grethe, A., Ozyurt, I.B., Busa, E., Quinn, B.T., Fennema-Notestine, C., Clark, C.P., Morris, S., Bondi, M.W., Jernigan, T.L., Dale, A.M., Brown, G.G., Fischl, B., 2007. A technique for the deidentification of structural brain MR images. Hum. Brain Mapp. 28, 892–903.

Dale, A.M., Fischl, B., Sereno, M.I., 1999. Cortical surface-based analysis. I. Segmentation and surface reconstruction. Neuroimage 9, 179–194.

Fischl, B., 2012. FreeSurfer. Neuroimage 62, 774–781.

Fischl, B., Salat, D.H., Busa, E., Albert, M., Dieterich, M., Haselgrove, C., van der Kouwe, A., Killiany, R., Kennedy, D., Klaveness, S., Montillo, A., Makris, N., Rosen, B., Dale, A.M., 2002. Whole brain segmentation: automated labeling of neuroanatomical structures in the human brain. Neuron 33, 341–355.

Fischl, B., Sereno, M.I., Dale, A.M., 1999. Cortical surface-based analysis. II: Inflation, flattening, and a surface-based coordinate system. Neuroimage 9, 195–207.

Jouvent, E., Reyes, S., Mangin, J.-F., Roca, P., Perrot, M., Thyreau, B., Hervé, D., Dichgans, M., Chabriat, H., 2011. Apathy is related to cortex morphology in CADASIL. A sulcal-based morphometry study. Neurology 76, 1472–1477.

Mills, K.L., Goddings, A.-L., Herting, M.M., Meuwese, R., Blakemore, S.-J., Crone, E.A., Dahl, R.E., Güroğlu, B., Raznahan, A., Sowell, E.R., Tamnes, C.K., 2016. Structural brain development between childhood and adulthood: Convergence across four longitudinal samples. Neuroimage 141, 273–281.

Ochiai, T., Grimault, S., Scavarda, D., Roch, G., Hori, T., Rivière, D., Mangin, J.F., Régis, J., 2004. Sulcal pattern and morphology of the superior temporal sulcus. Neuroimage 22, 706–719.

Pardoe, H.R., Kucharsky Hiess, R., Kuzniecky, R., 2016. Motion and morphometry in clinical and nonclinical populations. Neuroimage 135, 177–185.

Perrot, M., Rivière, D., Mangin, J.-F., 2011. Cortical sulci recognition and spatial normalization. Med. Image Anal. 15, 529–550.

Rosas, H.D., Liu, A.K., Hersch, S., Glessner, M., Ferrante, R.J., Salat, D.H., van der Kouwe, A., Jenkins, B.G., Dale, A.M., Fischl, B., 2002. Regional and progressive thinning of the cortical ribbon in Huntington’s disease. Neurology 58, 695–701.

Rosen, A.F.G., Roalf, D.R., Ruparel, K., Blake, J., Seelaus, K., Villa, L.P., Ciric, R., Cook, P.A., Davatzikos, C., Elliott, M.A., Garcia de La Garza, A., Gennatas, E.D., Quarmley, M., Schmitt, J.E., Shinohara, R.T., Tisdall, M.D., Craddock, R.C., Gur, R.E., Gur, R.C., Satterthwaite, T.D., 2018. Quantitative assessment of structural image quality. Neuroimage 169, 407–418.

Satterthwaite, T.D., Wolf, D.H., Loughead, J., Ruparel, K., Elliott, M.A., Hakonarson, H., Gur, R.C., Gur, R.E., 2012. Impact of in-scanner head motion on multiple measures of functional connectivity: relevance for studies of neurodevelopment in youth. Neuroimage 60, 623–632.

White, T., Su, S., Schmidt, M., Kao, C.-Y., Sapiro, G., 2010. The development of gyrification in childhood and adolescence. Brain Cogn. 72, 36–45.
